# The Enzymatic and Non-Enzymatic Antioxidant System Response of the Seagrass *Cymodocea nodosa* to Bisphenol-A Toxicity

**DOI:** 10.3390/ijms23031348

**Published:** 2022-01-25

**Authors:** Paraskevi Malea, Danae Kokkinidi, Alkistis Kevrekidou, Ioannis-Dimosthenis S. Adamakis

**Affiliations:** 1 School of Biology, Department of Botany, Aristotle University of Thessaloniki, 54124 Thessaloniki, Greece; danaekokkinidi@gmail.com; 2 School of Engineering, Department of Chemical Engineering, Aristotle University of Thessaloniki, 54124 Thessaloniki, Greece; alkistiskebrek@gmail.com; 3 Section of Botany, Department of Biology, National and Kapodistrian University of Athens, 15784 Athens, Greece; iadamaki@biol.uoa.gr

**Keywords:** ascorbate peroxidase activity, BPA, hydrogen peroxide, lipid peroxidation, phenolic compounds, superoxide dismutase activity, marine angiosperm

## Abstract

The effects of environmentally relevant bisphenol A (BPA) concentrations (0.3, 1 and 3 μg L^−1^) were tested at 2, 4, 6 and 8 days, on intermediate leaves, of the seagrass *Cymodocea nodosa*. Hydrogen peroxide (H_2_O_2_) production, lipid peroxidation, protein, phenolic content and antioxidant enzyme activities were investigated. Increased H_2_O_2_ formation was detected even at the lowest BPA treatments from the beginning of the experiment and both the enzymatic and non-enzymatic antioxidant defense mechanisms were activated upon application of BPA. Elevated H_2_O_2_ levels that were detected as a response to increasing BPA concentrations and incubation time, led to the decrease of protein content on the 4th day even at the two lower BPA concentrations, and to the increase of the lipid peroxidation at the highest concentration. However, on the 6th day of BPA exposure, protein content did not differ from the control, indicating the ability of both the enzymatic and non-enzymatic mechanisms (such as superoxide dismutase (SOD) and phenolics) to counteract the BPA-derived oxidative stress. The early response of the protein content determined that the Low Effect Concentration (LOEC) of BPA is 0.3 μg L^−1^ and that the protein content meets the requirements to be considered as a possible early warning “biomarker” for *C. nodosa* against BPA toxicity.

## 1. Introduction

The ever-increasing leaches from plastic pollution, have unavoidably led to some endocrine disrupting chemicals (EDCs) to be ubiquitous in nearly all the aquatic environments [1,2]. EDC compounds have detrimental effects on several organisms, since they disrupt the hormonal equilibrium. The most common EDC is bisphenol A (2,2-bis[4 -hydroxyphenyl]propane; BPA). This plasticizer is an organic chemical used as an intermediate in the production of numerous plastic utensils [3]. Due to the increasing demand for the aformentioned products, BPA production has constantly grown in the last years. For instance, the global demand was 5.5 million tons in 2011 [4] and it was expected to have grown at a rate of 4.6% per year the period from 2013 to 2019 [4]. BPA discharges which enter into coastal systems via rivers or rain [5], have steadily increased the BPA concentrations found in river waters (0.014–21.0 μg L^−1^)*,* in seawater (<0.0005–2.470 μg L^−1^), in wastewater plant effluents (0.087–5.625 μg L^−1^) and in industrial wastewater treatment wastes (1–150 μg L^−1^) [6,7]. Thus, BPA leaching into the aquatic environment represents a potential risk [2]. Therefore, extensive toxicity studies concerning its impacts on aquatic organisms have been conducted mainly focusing on animals (i.e., fish, amphibians, crustaceans and mollusks) [8,9] and microalgae [10], where the effects of higher than the environmentally relevant concentrations were investigated. BPA endocrine disruption effects were detected even at the concentrations below 1 μg m^−3^ [11], and the scarcity of data concerning BPA impact on aquatic organisms is particularly alarming [2] since, BPA disrupts a broad range of biological functions [12]. For example in marine, freshwater and terrestrial higher plants previous studies showed that BPA exposure hinders their growth and development by: (i) inducing oxidative stress and activating the antioxidant system, (ii) interfering with/or modifying their photosynthetic activity (chlorophyll fluorescence, photosynthetic pigments), (iii) altering the total amount of soluble sugar and starch, (iv) disrupting the hormone synthesis, (v) damaging the cell cytoskeleton and (vi) hindering cell division and subsequently inhibiting various plant part elongation [13,14,15,16,17,18,19,20,21,22,23,24,25].

One interesting route of BPA toxicity is the production of reactive oxygen species (ROS) (superoxide, O_2_^−^, hydrogen peroxide, H_2_O_2_, singlet oxygen ^1^O_2_) [14,22,23,26]. ROS overproduction can interact with cell membrane proteins and phospholipids, leading to lipid peroxidation [14,17,26], can activate the antioxidant system (antioxidant enzymes, i.e., superoxide dismutase, ascorbate peroxidase, catalase) [14,26] or induce the production of the antioxidant non-enzymatic substances (i.e., secondary metabolites, phenolic compounds, etc.) [16,23].

Aquatic plants could be used as bioindicators of BPA toxicity [27]. In this sense, *Cymodocea* *nodosa* (Ucria) Ascherson 1870, one of the five most widespread Mediterranean seagrass species [28] and belonging to the taxonomic families required for the determination of a Species Sensitivity Distribution (SSD) (EU Technical Guidance Document of the European Union (EU), 2003) [29], can act as one BPA “bioindicator” organism [25]. *C. nodosa* has the ability to act as a “bioindicator” organism of many anthropogenic-derived pressures [30], such as metals or metallic nanoparticles [31,32,33,34,35] and some cellular and physiological ‘markers’ were determined, for studying the impact of these pollutants [25,31,32,33,34,35]. Moreover, biotic indexes, in *C. nodosa*, for the ecological status assessment of coastal and transitional waters have also been established [36,37].

Concerning the impact of BPA to seagrasses, the only relevant data existing is that reported for *C. nodosa* plants [20,23,25]. It was demonstrated that the BPA-induced damages on *C. nodosa* cell cytoskeleton (actin filaments and microtubules) and the inhibition of various plant part elongation [20,25] act as BPA-induced stress “biomarkers”. The BPA toxicity mechanism on *C. nodosa* leaf photosynthetic machinery was further investigated. It has been stated that BPA-derived H_2_O_2_ generation (evaluated by positive H_2_DCF-DA staining) seemed to be a key factor, triggering oxidative damages on epidermal leaf cells but simultaneously inducing retrograde signaling, also conferring tolerance to BPA [23]. However, in these previous studies the effects of BPA on the enzymatic antioxidant system were not investigated. Therefore, in this study, in BPA-treated (0.3, 1 and 3 μg L^−1^) *C. nodosa* intermediate leaf blades, ROS generation (H_2_O_2_), lipid peroxidation, protein and phenolic content, and antioxidant enzyme activity systems were investigated. The main goal of this study was to assess the impact of environmentally relevant BPA concentrations [8] on *C. nodosa* leaf blade oxidative stress parameters (reactive oxygen species, antioxidant enzymes, phenolic compounds), and the alterations in the cell membrane permeability (lipid peroxidation) and their protein contents. In addition, the toxic BPA effect levels were evaluated, and the possible sensitive stress “biomarkers” were identified as early warning indicators of BPA risk in aquatic environment.

## 2. Results

### 2.1. Effects of BPA on Oxidative Stress Parameters

#### 2.1.1. Hydrogen Peroxide Production

**Figure 1:**
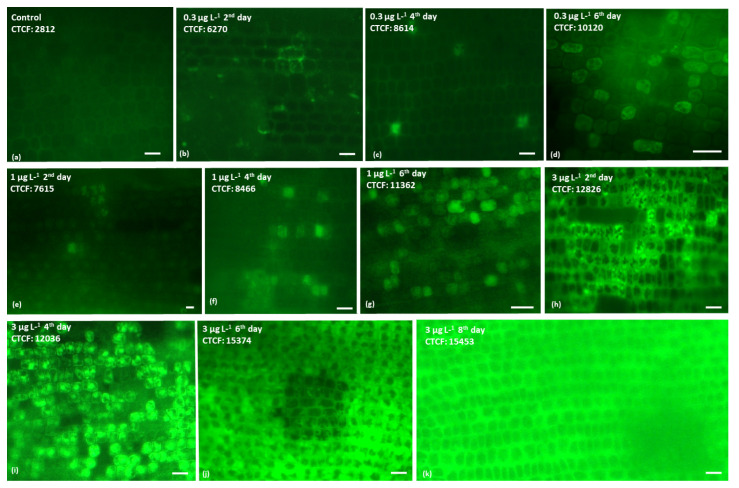
Indicative fluorescence images of hydrogen peroxide (H_2_O_2_) after H_2_DCF-DA staining in *Cymodocea nodosa* intermediate leaves, at control (**a**), 0.3 (**b**–**d**), 1 (**e**–**g**) and 3 (**h**–**k**) μg L^−1^ BPA treatments, on 2, 4, 6 and 8 days. The corrected total cell fluorescence (CTCF) values are depicted on the images. Scale bars: 50 μm.

Levels of H_2_O_2_ in the epidermal cells of intermediate *C. nodosa* leaves increased with increasing incubation time and BPA concentrations, as indicated by the H_2_DCF-DA fluorescence (Figure 1). H_2_O_2_ was detected even at the lowest BPA treatment (0.3 μg L^−1^) from the beginning of the experiment (Figure 1b). H_2_O_2_ production, expressed as the mean CTCF (Corrected Total Cell Fluorescence) values, which is calculated at all BPA treatments and each incubation time, was significantly higher than in the control (Mann Whitney U-test, *p* < 0.001) (Table 1).

**Figure 2:**
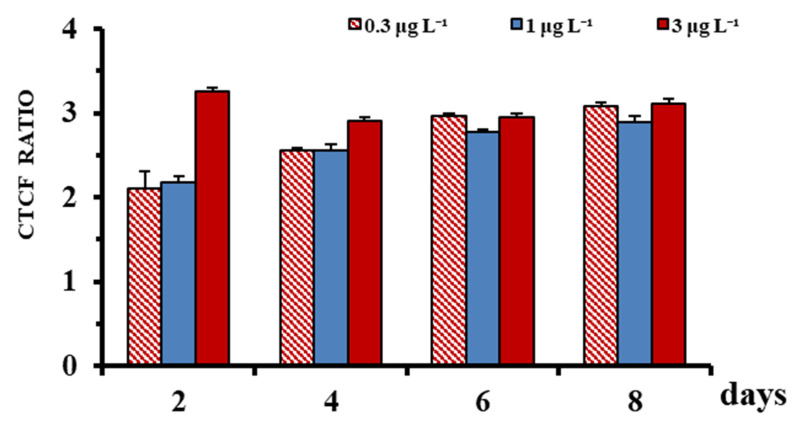
Time course of endogenous H_2_O_2_ detected in *C. nodosa*, at 0.3, 1 and 3 μg L^−1^ BPA treatments expressed as the ratio of corrected total cell fluorescence (CTCF) values in treated to that in the untreated leaves; mean CTCF values (±SE) were derived from 54 CTCF values per concentration and time.

The ratio of the CTCF values in BPA-treated leaves to their values in the control was, generally increased by increasing incubation time, and upon BPA treatments, mainly on the first incubation days (2–4 days) (Figure 2). However, the highest H_2_O_2_ generation (max CTCF ratio) was observed at the 3 μg L^−1^ in the beginning of the experiment (Figure 2).

#### 2.1.2. BPA Effects on Antioxidant Enzyme Activity, Total Protein Content and Lipid Peroxidation

**Figure 3:**
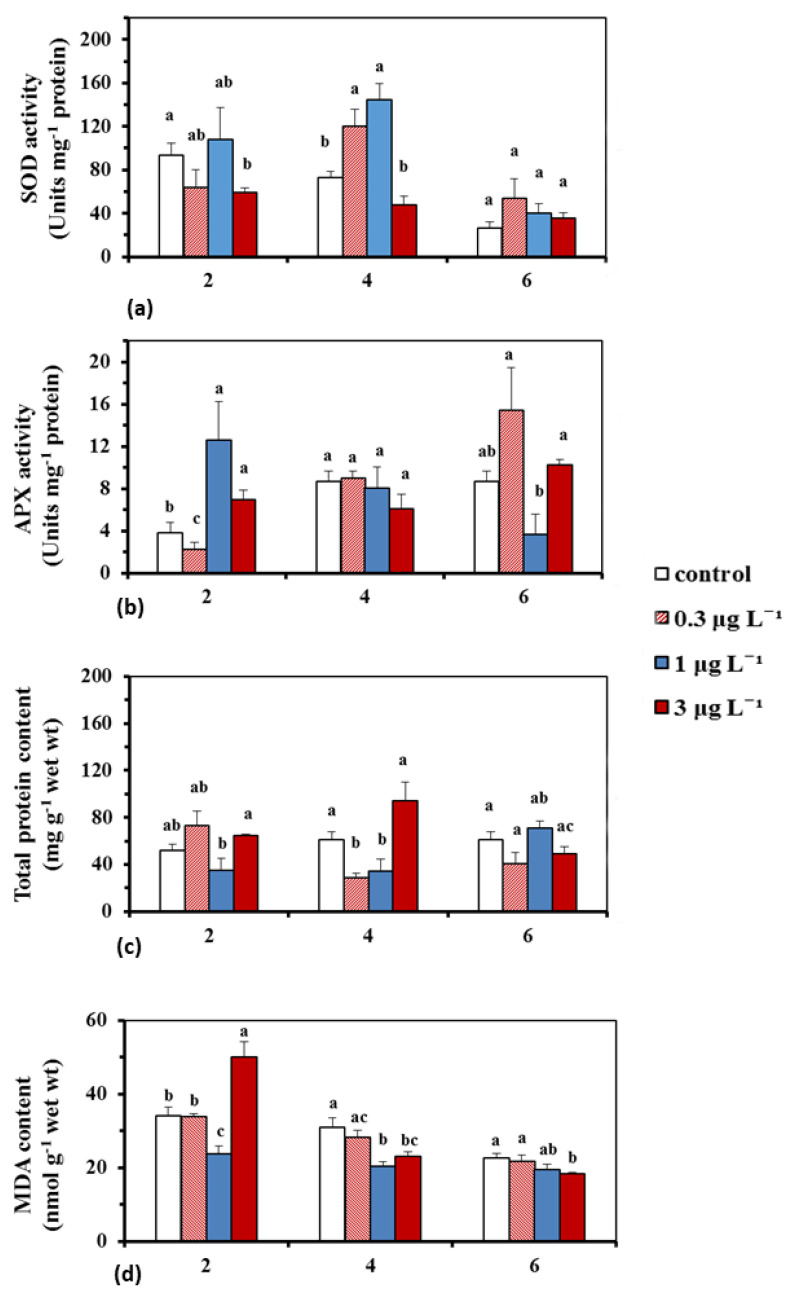
Changes in SOD activity (**a**), APX activity (**b**), total protein content (**c**), and MDA content (**d**) in *Cymodocea nodosa* intermediate leaves, at 0.3, 1 and 3 μg L^−1^ BPA treatments and in the control, exposed for 2, 4 and 6 days; mean ± SE from three subsamples; different letters express significantly different values between treatments and the control and between treatments (Mann–Whitney U–test; *p* < 0.05).

Superoxide dismutase activity (SOD) at the two lower BPA solutions (0.3 and 1 μg L^−1^), on the 4th day, was significantly (*p* < 0.05) increased compared to the control (1.6–2.0-fold) and the higher solution (3 μg L^−1^) and continued to be slightly elevated (*p* > 0.05) on the 6th day. At the highest treatment (3 μg L^−1^), SOD activity was equal to (*p* > 0.05) or lower (*p* < 0.05) than the control and the two lower treatments in the experiment (Figure 3a).

Ascorbate peroxidase (APX) activity at 1 and 3 μg L^−1^ was higher (*p* < 0.05) compared to the control and the lowest BPA treatment (0.3 μg L^−1^), at the beginning of the experiment (2nd day), whereas at 0.3 μg L^−1^ BPA, its activity was elevated (*p* < 0.05) relative to 1 μg L^−1^ on the 6th day (Figure 3b).

Protein content at 1 μg L^−1^ BPA was lower (*p* < 0.05) than the 3 μg L^−1^ on the 2nd day, and at the 0.3 and 1 μg L^−1^ BPA treatments, it was lower (*p* < 0.05) than the control (*p* < 0.05) and the 3 μg L^−1^ BPA treatment on the 4th day (Figure 3c).

Lipid peroxidation, as expressed by MDA (Malondialdehyde) content, at the 3 μg L^−1^ was higher (*p* < 0.05) than the control and the two lower BPA treatments, at the start of the experiment, whereas it was lower (*p* < 0.05) than the control and the lowest BPA solution on the 4th and 6th day (Figure 3d).

#### 2.1.3. Total Phenolic Content

**Figure 4:**
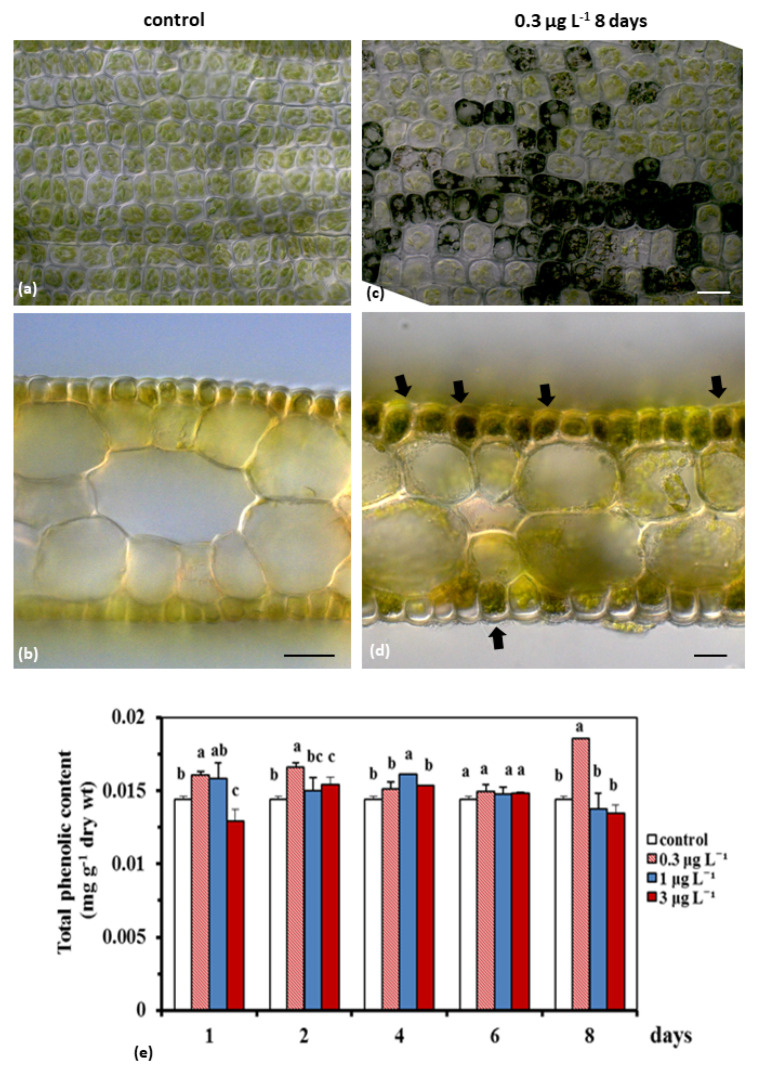
Indicative images of DIC micrographs of control (**a**,**b**) and BPA-treated (0.3 μg L^−1^ 1 for 8 days) (**c**,**d**) *Cymodocea nodosa* leaves. Under BPA effect, epidermal cells with dark content appeared (black arrows) as indicated in both surface (**a**,**c**), and cross (**b**,**d**) leaf sections. Changes in total phenolic content in *Cymodocea nodosa* leaves, at 0.3, 1 and 3 μg L^−1^ BPA treatments and in the control, exposed for 1, 2, 4, 6 and 8 days; mean ± SE from three subsamples (**e**). Different letters express significantly different values between treatments and the control and between treatments (Mann–Whitney U–test; *p* < 0.05).

Leaf epidermal cells under BPA treatment are filled with dark (probably phenolic material) (Figure 4a–d). Mann-Whitney U-test showed that the total phenolic content at the lowest treatment (0.3 μg L^−1^) was elevated (*p* < 0.05) compared to the control and the highest solution from the beginning (1st and 2nd day) and at the end of the experiment (8th day) (Figure 4e). At 1 μg L^−1^ the phenolic compound production also started from the beginning of the test (1st and 2nd day), but this increase was significant (*p* < 0.05) on the 4th day (Figure 4e). Finally, at the 3 μg L^−1^ BPA, their values increased relative to the control, mainly on the 2nd day (*p* < 0.05), and on the 4th day (*p* > 0.05) as well (Figure 4e).

## 3. Discussion

Laboratory toxicity data concerning BPA effects on aquatic macrophytes are scarce and have been conducted on a limited number of species. For instance, BPA effects were addressed on the freshwater angiosperms *Lemna gibba* and *Ceratopyllum demersum* and the seagrass *Cymodocea nodosa*. In *L. gibba*, at higher than the environmentally relevant concentrations, BPA affected its growth and survival, photosynthetic activity (chlorophyll fluorescence, photosynthetic pigments), and sugar and starch contents [16,38] and caused a severe cellular damage as indicated by the increase MDA levels [15]. Similarly, in *C. demersum*, BPA caused protein and photosynthetic pigment reductions [39]. BPA concentrations applied in our experiments (0.3, 1 and 3 μg L^−1^) were within the environmentally relevant concentrations (0.08–12.5 μg L^−1^; [7,8]). Specifically, 0.3 and 1 μg L^−1^ were among those found in seawater and 3 μg L^−1^ was in the range of those in surface waters and emitted from wastewater treatment plants [6,7,8]. Therefore, our study further consolidates the notion that BPA, even at low concentrations, is detrimental to aquatic organisms [3], including *C. nodosa* [20,23,25], as discussed below.

Limited ecotoxicological studies exist addressing the effects of BPA on seagrasses, and these are limited on *C. nodosa* [20,23,25]. However, in these studies the antioxidant mechanisms, the alterations in the cell membrane permeability and the protein content, have not been investigated. In *C. nodosa*, BPA (i) disrupted the cell cytoskeleton (actin filament, AF and microtubules, MT) at 0.03 and 0.1 μg L^−1^ BPA, respectively [20], (ii) retarded growth of juvenile leaf blades, adult leaves and plagiotropic rhizome internodes (10-day EC_50_ values: 0.137, 0.029 and 0.025 μg L^−1^, respectively [25]), and (iii) affected PSII function probably via the H_2_O_2_ increase (positive H_2_DCF-DA staining) firstly at 0.5 μg L^−1^ BPA exposure [23]. Under BPA exposure the disruption of the electron transfer between the PSII and PSI reaction centers on aquatic microalgae and terrestrial plants [22,40,41,42] resulted in ROS (superoxide, O^2−^, hydrogen peroxide, H_2_O_2_, etc.) generation [22,26,43]. In a previous study, in *C. nodosa*, under BPA exposure, a decrease of the photosynthetic parameters Φ*PSII* and Φ*NPQ*, and an increase of Φ*NO* were observed, which supported the role of H_2_O_2_ in the *C. nodosa* response against BPA toxicity [23]. In this study, in *C. nodosa* intermediate leaf blades, BPA induced H_2_O_2_ overproduction even at the lowest concentrations used (0.3 μg L^−1^), and the shorter duration of exposure (Figure 1 and Figure 2; Table 1) (see also [23]). At 3 μg L^−1^, H_2_O_2_ generation was elevated compared to the control and the other treatments, mainly the first 2–4 days of BPA treatment (Figure 1 and Figure 2; Table 1). It has been stated that, up-regulated H_2_O_2_ production could cause both oxidative damages, but can also trigger retrograde signaling, conferring BPA-tolerance [23], possibly by activating the antioxidant machinery. Oxidative-stress related parameters (both enzymatic and non-enzymatic antioxidant parameters) in marine flora species have been found to change as a response against various stressors, [34,35,44,45,46,47,48]. Likewise, in *C. nodosa* BPA is a stressor that induces ROS production, especially H_2_O_2_ (this study; [23]). H_2_O_2_ is a parameter considered as an early marker of various stressors (i.e., [49]).

H_2_O_2_ is relatively stable and a compound with high longevity [50]. Therefore, when H_2_O_2_ overproduction exceeds the antioxidant defense capacity can create oxidative stress in mitochondria, chloroplasts and peroxisomes [51], and can activate protective mechanisms, including the enzymatic antioxidant system (e.g., SOD, APX, CAT) [52]. In *C. nodosa* leaf blades the activity of SOD, an enzyme which catalyzes the dismutation of O^2−^ radical to H_2_O_2_ and O_2_ (e.g., [53]), was increased compared to the control at the two lowest treatments (0.3 and 1 μg L^−1^) on the 4th and up to 6th day, following an increase of H_2_O_2_ production pattern (see CTCF ratio). In contrast, at the highest treatment concentration (3 μg L^−1^), SOD activity was lower or equal compared to the control and the other BPA solutions used (Figure 3a). The observed inhibition of SOD activity at high BPA concentrations, despite the H_2_O_2_ generation, was also confirmed by other studies (e.g., [26], in *Oryza sativa* leaves), and could be attributed to the fact that high BPA doses are able to overwhelm the self-defense system, resulting in functional and structural cell damages, which seems to be also the case in *C. nodosa* leaves, since BPA damaged the structural integrity of chloroplasts, dictyosomes and endoplasmic reticulum [23]. APX activity, which has the highest affinity to convert H_2_O_2_ to H_2_O [54], was activated by BPA, primary, at 1 and 3 μg L^−1^ on the beginning of the experiment and at 0.3 μg L^−1^ on the 6th day (Figure 3b), following the increase of H_2_O_2_ production (Figure 1). The possible dissimilarity among variations of ROS production and APX activity (Figure 1 and Figure 3b) could be attributed to the fact that, other enzymes (i.e., catalase, CAT), which share the same substrate (H_2_O_2_) could be possibly activated. For instance, in a previous study CAT and APX activities showed dissimilar trends, due to the unavailability of their common substrate [55].

Lipid peroxidation of polyunsaturated fatty acids is revealed by MDA formation [56]. The endogenous H_2_O_2_ BPA-derived increase could trigger MDA creation, a phenomenon recorded in both aquatic and terrestrial flora under BPA toxicity [13,43,46,57]. The overproduction of H_2_O_2_ in *C. nodosa* leaf blades at 3 μg L^−1^ on the 2nd day (maximum value of CTCF ratio) (Figure 1), indicated the inability of APX, which also increased on this day compared to the control, to successfully scavenge H_2_O_2_ production, as the membrane lipid peroxidation increased (maximum MDA values) (Figure 3d). The decrease of MDA on the other incubation days, at this treatment could be attributed to the decrease of H_2_O_2_ (Figure 1, Figure 2 and Figure 3d) or/and to a disruption of enzymatic defense mechanism due to structural and functional cell injuries [23].

Protein contents decreased in marine diatoms with the application of BPA [58], in the model plant *Arabidopsis thaliana* [59] and in the soybean plants [60], in a time and concentration related manner, with the BPA-derived oxidative stress being the reason of the observed protein content decrease. Likewise, the reduction in *C. nodosa* leaf protein content, mainly at the two lowest treatments on the 4th day and at 1 μg L^−1^ on the 2nd day (Figure 3c), may indicate an inhibition of protein biosynthesis or acceleration of protein decomposition [60], due to oxidation, even from the beginning of the experiments (Figure 1 and Figure 2). This early response of the protein content (even from the 4th day), at the two lowest environmentally relevant BPA concentrations, determines that the LOEC value (Low Effect Concentration) is 0.3 μg L^−1^, a LOEC value consistent with what is already reported, based on other markers [25].

Polyphenols are an important class of specialized metabolites that play crucial physiological roles throughout the plant life cycle, including responses to stress. It is well known that phenolic biosynthetic pathways are usually activated under harmful environmental conditions, such as drought, extreme temperatures, salinity, metal pollution, and ultraviolet radiations [61]. Secondary metabolites, such as phenolic compounds, were considered suitable “biomarkers” against abiotic and biotic stress in marine angiosperms, which contributed to their antioxidant activities [44,62,63]. BPA seems to be a factor inducing phenolic compound increase (Figure 3). The rise in the phenolic compounds at 0.3 μg L^−1^ BPA-treated *C. nodosa* leaves, compared to the control, even from the beginning of the experiment (1–2 days), which was then overstimulated at the end of the experiment (8th day), may indicate an activation of an antioxidant protective mechanism (see elevated CTCF ratio; Figure 2); this is also obvious for 3 μg L^−1^ BPA, on the 2nd experimental day.

Biochemical parameters (e.g., phenolics and proteins) are highly dynamic and variable among seagrasses, as they are modulated by external environmental or internal cellular factors [64]. However, biochemical “biomarkers” especially proteome-related ones recently have been considered as a promising tool in ecotoxicological research [65]. In our experiments on the 4th day, the stimulation of the enzymatic antioxidant mechanism (see high SOD activity in 0.3 and 1 μg L^−1^ BPA treatments) and the non-enzymatic mechanism (see high phenolic content at 1 μg L^−1^ on 4th day) may mitigate the overproduction of H_2_O_2_ and lead to the recovery of SOD activity, and of protein and phenolic contents, on the 6th day, at the control levels (Figure 3c and Figure 4, respectively). The early change in the protein levels was detected, and since most “biomarkers” are proteins (biotransformation enzymes, oxidative stress related proteins, cell death related proteins etc.) [66], it could help to conclude that protein levels could meet the requirements to be an early warning “biomarker” of BPA toxicity on *C. nodosa*.

## 4. Materials and Methods

### 4.1. Plant Collection and Experimental Conditions

The seagrass *Cymodocea nodosa* (Ucria) Ascherson 1870 collected, during June from the eastern coast of the Gulf of Thessaloniki, Northern Aegean Sea (40°24′24.03″ N, 22°53′43.21″ E), were kept for 24 h in seawater under laboratory conditions (16/8 h day/night regime, ambient temperature 21 ± 1 °C, irradiance of 120 μmοl m^−2^ s^−1^) to acclimatize. Seawater used in the experiment was also collected from the sampling area and had a BPA concentration below detection limit (DL = 10 ng L^−1^) [25].

Seagrass consisting of horizontal rhizome, vertical rhizomes, roots and leaf shoots were incubated in non-BPA-based polypropylene (PP) copolymer (PPCO) constantly aerated aquaria, containing 20 L of 0.3, 1 and 3 μg L^−1^ bisphenol-A (BPA; Sigma, Taufkirchen, Germany) solutions in filtered seawater (0.45 μm Whatman GF/C) and filter seawater (used as a control). It should be noted that attention was paid to the selection of leaf shoots without epiphytes, while only the first intermediate leaves were used for our experiments, as the adult leaves are usually covered with epiphytes. BPA solutions were changed every two days, to adjust to the original levels out of an abundance of caution, although BPA in seawater remains stable for 35 days [67]. The experiments were run in triplicates. From each BPA solution and the control after 1, 2, 4, 6 and 8 days, blades of the first intermediate leaf [31] with nearly the same size (mean leaf length ± SE: 86 ± 12.3 mm) from at least six shoots per dose and day (two per aquarium) were randomly removed for H_2_O_2_ production measurements. Additionally, each day from each treatment, intermediate leaves from another nine shoots (three per aquarium) were selected for the estimation of antioxidant enzyme activities and protein content, and another nine shoots for the estimation of lipid peroxidation, and other nine shoots for the estimation of the total phenolic compound contents.

### 4.2. Hydrogen Peroxide Production

BPA treated and untreated intermediate leaf blades at each exposure time were incubated with 2′, 7′-dichlorofluoresce in diacetate (H_2_DCF-DA, Sigma) in dimethyl sulfoxide (DMSO) [34]. Τhe observation was made under a Zeis AxioImager Z.2 fluorescence microscope equipped with an MRc5 Axiocam. The corrected total cell fluorescence (CTCF) was measured, with the Image J software using the equation: CTCF = integrated density—(area of selected cell * mean fluorescence of background readings); CTCF values were attained for the tip, middle and basal leaf part, totally 54 CTCF values (3 areas per leaf part × 3 parts × 6 leaves) per concentration and day and the mean CTCF ratio, as the mean ratio of CTCF values in treated to that in the un-treated leaves was also calculated [49].

### 4.3. Estimation of Antioxidant Enzyme Activities, Total Protein and MDA Content

At each BPA concentration at the 2nd, 4th and 6th day, three subsamples were implemented. Each subsample (3 leaves per subsample, 100 mg wet weight) were grounded in liquid nitrogen and afterwards homogenized in 3 mL of 50 mM sodium phosphate buffer (pH 7.8), containing 0.1 mM EDTA and 2% (*w*/*v*) polyvinyl pyrrolidone (PVPP). The homogenates were centrifuged at 16.500× *g* for 30 min at 4 °C. Total protein content (mg g^−1^ wet weight) was determined in the supernatants, based on the principle of protein-dye binding (Coomassie Brilliant Blue G-250), using bovine serum albumin (BSA) as standard [68].

For the quantification of SOD activity, a reaction mixture (5 mL) of 50 mM potassium phosphate, 0.1 mM EDTA, TritonX-100 0.025% (pH 7.8), 13 mM methionine and 0.075 mM nitro blue tetrazolium (NBT) were used; in aliquots (50 μL) of the extracts 0.002 mM riboflavin was added. SOD activity (Units mg^−1^ protein) was determined under UV light at 560 nm, based on the inhibition rate of nitro blue tetrazolium (NBT) to photochemical decline [69].

APX activity was estimated according to a modified method [69]. Three subsamples (3 × 100 mg wet weight) were incubated in the reaction mixture (5 mL) consisting of 50 mM potassium phosphate, 0.1 mM EDTA (pH 7.8), 0.5 mM ascorbic acid, an aliquot (50 μL) of the supernatant and 0.1 mM H_2_O_2_. The alteration in absorbance was monitored at 290 nm for 1 min. APX activity (Units mg^−1^ protein) was measured using the extinction coefficient 2.8 mM^−1^ cm^−1^.

MDA content was determined according to the method described in [70], in triplicate. Three subsamples (3 × 100 mg wet weight) were grounded in liquid nitrogen and homogenized after the addition of 0.1% trichloroacetic acid (TCA) and centrifuged at 15.000× *g* for 10 min, at 4 °C. The supernatants (1.25 mL) were combined with 3.75 mL of 0.5% thiobarbituric acid (TBA), diluted in 20% TCA, heated at 95 °C for 30 min, and centrifuged for 3 min. MDA concentration (nmol g^−1^ wet weight) was measured spectrophotometrically (Camspec M501, Single Beam Scanning, UV/Visible, Shimadzu, Tokyo, Japan) at 532 and 600 nm, using the extinction coefficient of 155 m^−1^ M^−1^ cm^−1^.

### 4.4. Total Phenolic Compound Content

Phenolic compounds were analyzed in 3 freeze dried subsamples [71] s (100 mg dry weight per subsample) per concentration and day. Total phenolic content (mg g^−1^ dry weight) was determined in the extracts, with a modified Folin-Ciocalteu method [72,73]. An aliquot of the extract (250 μL) was combined with 2.5 mL Folin-Ciocalteu reagent (previously diluted with water 1:10 *v*/*v*), 250 μL distilled water and 2 mL sodium carbonate 0.7 M. The mixture was incubated in a water-bath for 30 min at 37 °C and the absorbance was measured at 760 nm with a spectrophotometer (PharmaSpec UV-1700, Shimadzu, Tokyo, Japan). Gallic acid was used as a standard. The Folin-Ciocaltefreu formulation, is known to be less affected by interfering compounds, and to avoid precipitation, give greater color, less variation and better recovery [71].

### 4.5. Microscopy

Leaf specimens or leaf cross sections were placed on microscope slides in sea water and observed either under a Zeiss AxioImager Z.2 or a Zeiss Axioplan microscope, both equipped with a MRc5 Axiocam (Zeiss, Berlin, Germany). Differential interference contrast (DIC) microscopy was applied, and imaging acquisition was performed with the AxioVision SE64 4.8.3 or the ZEN. Blue 2.3. software following the manufacturer’s instructions as already reported [23].

### 4.6. Data Analysis

Analyses on raw and ln-transformed data indicated unequal variances and severe violation of the normality assumption. Mann-Whitney U-test analysis was carried out to determine differences in the response parameters among BPA concentrations and between BPA concentrations and the control at each incubation time; these approaches consider average values, 54 values for CTCF, and for enzyme activity, MDA, and protein contents, the three subsamples at each BPA concentration and time. The above statistical analysis was carried out using IBM SPSS^®^25.0.

## 5. Conclusions

In summary, our results demonstrated that H_2_O_2_ formation was detected in intermediate *C. nodosa* leaves even at the lowest BPA treatment, from the beginning of the experiment, causing the antioxidant defense mechanisms to be activated, even at environmentally relevant concentrations. ROS overproduction resulted in protein content decline at the two lower BPA exposures, on the 4th day and lipid peroxidation, at 3 μg L^−1^ of BPA, at the beginning of the test. The enzymatic and non-enzymatic antioxidant defense mechanism (i.e., SOD and phenolic compounds) effectively scavenges excess ROS, on the 4th day, re-establishing protein content to control levels on the 6th day. Therefore, this early warning response of the protein content determines the LOEC of BPA to be 0.3 μg L^−1^, and additionally, it meets the requirements for protein content to be considered as a possible early warning ‘biomarker’ for *C. nodosa* against BPA toxicity. However, it should be noted that biochemical parameters in ecotoxicological studies should be studied in combination as they are interrelated.

## Figures and Tables

**Table 1:** CTCF values (Corrected Total Cell Fluorescence) (mean ± SE) in BPA treated (0.3, 1 and 3 μg L^−1^) *Cymodocea nodosa* intermediate leaves and in the Control (C), after 2, 4, 6 and 8 days. Comparison (Mann-Whitney U-test) of the mean CTCF values at each BPA treatment and each incubation time with the respective values in the control is also given. N = 54 values per concentration and time.

	BPA Concentrations (μg L^−1^)				Comparison of CTCF Values		
Days	Control	0.3	1	3	C-0.3	C-1	C-3
2	3571.2 ± 78.7	6808.3 ± 103.5	7795.3 ± 261.3	11,638.8 ± 152.7	***	***	***
4	3583.5 ± 76.7	9153.5 ± 104.5	9140.3 ± 261.3	10,404.8 ± 150.7	***	***	***
6	3505.5 ± 75.7	10,388.3 ± 102.5	9712.1 ± 121.8	10,402.1 ± 155.7	***	***	***
8	3437.6 ± 78.7	10,445.2 ± 104.5	9767.0 ± 121.8	10,481.1 ± 151.6	***	***	***

Mann-Whitney U-test, ***: *p* < 0.001.

## Data Availability

The data presented in this study are available in this article.
